# Exploring Pathway-Based Group Lasso for Cancer Survival Analysis: A Special Case of Multi-Task Learning

**DOI:** 10.3389/fgene.2021.771301

**Published:** 2021-11-29

**Authors:** Gabriela Malenová, Daniel Rowson, Valentina Boeva

**Affiliations:** ^1^ Department of Computer Science, Institute for Machine Learning, ETH Zurich, Zürich, Switzerland; ^2^ Swiss Institute for Bioinformatics (SIB), Zürich, Switzerland; ^3^ Institut Cochin, Inserm U1016, CNRS UMR 8104, Université de Paris UMR-S1016, Paris, France

**Keywords:** survival analysis, Cox model, cancer, lasso, group lasso, multi-task, signalling pathways

## Abstract

**Motivation:** The Cox proportional hazard models are widely used in the study of cancer survival. However, these models often meet challenges such as the large number of features and small sample sizes of cancer data sets. While this issue can be partially solved by applying regularization techniques such as lasso, the models still suffer from unsatisfactory predictive power and low stability.

**Methods:** Here, we investigated two methods to improve survival models. Firstly, we leveraged the biological knowledge that groups of genes act together in pathways and regularized both at the group and gene level using latent group lasso penalty term. Secondly, we designed and applied a multi-task learning penalty that allowed us leveraging the relationship between survival models for different cancers.

**Results:** We observed modest improvements over the simple lasso model with the inclusion of latent group lasso penalty for six of the 16 cancer types tested. The addition of a multi-task penalty, which penalized coefficients in pairs of cancers from diverging too greatly, significantly improved accuracy for a single cancer, lung squamous cell carcinoma, while having minimal effect on other cancer types.

**Conclusion:** While the use of pathway information and multi-tasking shows some promise, these methods do not provide a substantial improvement when compared with standard methods.

## 1 Introduction

Survival analysis is an important topic in cancer research as it allows predicting the time to death or tumor progression as well as providing potential insights into the drivers of the disease. To predict the prognostic score of cancer patients, numerous survival models using patients’ molecular and clinical data have been proposed. In particular, gene expression data have been widely used since changes in the regulation of genes are ubiquitous in cancer. A variety of learning methods has been applied to survival data, *e.g.*, the Cox proportional hazard model, deep learning or random forests–see Matsuo et al. (2019) for their comparison on cervical cancer data. Going beyond just gene expression, these models have been used with many data types, such as radiography data and histopathology images, to investigate cancer survival (Wulczyn et al. (2020); Le et al. (2021)).

In this work, we utilized a version of the Cox model (Cox (1972))–its main strenghts being the ease of use, strong results and interpretability. While the deep learning approach has shown minor concordance improvements compared with the linear Cox model it suffers in terms of interpretability (Huang et al. (2020)), and random survival forests have consistently underperformed the linear models, although variants such as block forest do show promise for multi-omics data (Matsuo et al. (2019); Herrmann et al. (2020); Huang et al. (2019, 2020)).

The large number of genes and the high multicollinearity found between them, coupled with low sample numbers makes overfitting a major issue. It is therefore desirable to identify a smaller set of genes determining the cancer progression and severity. For this purpose, the Cox proportional hazard model can be supplemented with a lasso regression term (Tibshirani (1997)). Depending on the strength of the lasso regularization, some of the gene coefficients are truncated, effectively making the model sparse. However, there is no guarantee that the genes that are included in the Cox model are truly more predictive than those whose contributions are truncated. Indeed, slight variations in the sample set can lead to large variations in the included genes. One potential way to alleviate this is by grouping the genes.

Often, genes are activated together in synchronized processes called signaling pathways (Parikh et al. (2010)), a potential solution to the multicollinearity problem is therefore to build a model that is sparse not on a gene level, but on a pathway level. Of particular interest to us are pathways that are downstream of known cancer drivers. To achieve this, a version of the group lasso penalty, grouping genes by pathway, has been proposed and applied to cancer data (Obozinski et al. (2011)). Group lasso regularization works by performing ridge (*L*_2_) regression on the components within a group and then performing lasso (*L*_1_) regression across the groups. This means that the lasso component of the regularization causes entire groups to be included or removed from the model as a whole, while the ridge component reduces some of the coefficients’ size within any group that is included.

The version of the group lasso penalty that we use in this paper is the latent group lasso penalty. This penalty deals with the issue present in the naïve group lasso implementation that if the same gene is included in two groups and model coefficients for one of those groups are set to zero, then the gene contribution will also be set to zero in the second group. Latent group lasso allows for genes that fall into multiple groups to have independent coefficients, while not biasing the model towards their inclusion (Obozinski et al. (2011)).

Since their introduction for cancer, group lasso approaches have been used a number of times in survival analysis (Kim et al. (2012); Wang et al. (2018)). For instance, group lasso was used to integrate multi-omics data at the gene level (Xie et al. (2019)). However, to the best of our knowledge, the application of pathway level latent group lasso to gene expression data for cancer survival has not been investigated for large cohorts of patients such as the Tumor Genome Atlas (TCGA).

Of note, in addition to group lasso, there exist other pathway based approaches; they however failed to demonstrate major improvements compared with standard lasso. Zheng *et al.*, using Gene Set Variation Analysis (GSVA) to reduce gene expression to pathway expression, showed no significant improvement over standard lasso (Zheng et al. (2020)). Our own preliminary work using pathway based dimension reduction via PCA and autoencoders also resulted in worse results compared with standard lasso and the latent group lasso method (results not shown).

One further challenge associated with cancer survival modelling is that while across all cancers the number of samples is quite large (over 10,000 in the TCGA data set), the number of samples for any single cancer type can be as low as 36. Unfortunately, the naïve solution to this, merely training multiple cancers all together, does not perform well for a few reasons. Firstly, while there are many similarities across cancers, there are also many differences and thus building a single model to describe survival across all cancers is not feasible. Secondly, the survival across different cancers varies greatly and therefore models trained on all cancers together often get good global results by discriminating samples by cancer type, essentially giving high hazard scores to low survival cancer types and visa-versa, while being very inaccurate on any individual cancer.

We would like to combine multiple cancers into a single model in such a way that the similarities between them can be leveraged. A number of multi-task approaches has been tested for survival analysis, including autoencoders and clustered learning. Furthermore a kernel based approach has been developed which incorporated pathways and multi-tasking, but showed no consistent improvements compared with the random forest and survival SVM models (Li et al. (2016); Dereli et al. (2019); Kim et al. (2020)).

Additionally, several extensions of the group lasso regularization were proposed in the literature: a multivariate sparse group lasso–a version generalized to multidimensional response variables and predictors (Li et al. (2015)), or the generalized elastic net (GELnet)–a penalty that admits general weigths on both individual and pair-wise feature levels (Sokolov et al. (2016)). Neither of the group lasso generalizations, however, took into account the possibly different scaling of various cancer solutions. Moreover, the weigths are set *a priori*, so a particular pathway cannot be decoupled during the optimization process in case it is predictive for one cancer but not for the other one.

In this work, we present a method which links cancers together by means of a coupling term in the loss function which penalizes the models for having diverging coefficients (Evgeniou et al. (2005); Görnitz et al. (2011)). The aim of this method is to allow individual cancer models to leverage the information from other cancers, while still allowing the coefficients of each cancer model to vary individually. Ideally, this will drive the inclusion of genes corresponding to pathways equally important for survival in two cancer types. In this work, this multicancer coupling term has been incorporated in addition to latent group lasso.

## 2 Methods

### 2.1 Data

In this study, we used clinical and gene expression data generated by the TCGA Research Network: https://www.cancer.gov/tcga (Tomczak et al. (2015)). For this work, we selected 30 cancer types. From these, the 16 cancers with over 300 samples were used for the comparison of latent group lasso with naïve lasso and all 30 were used in the multi-tasking study. For each cancer, RNA-Seq data, time since inclusion in study, and survival status were used. The TCGA RNA-Seq data set was generated following the Firehose pipeline: MapSplice followed by RSEM (Li and Dewey (2011)), then normalized using upper quartile fragments per kilobase per million reads (FPKM-UQ).

The following cancer types were selected: Adrenocortical Carcinoma (ACC), Bladder Urothelial Carcinoma (BLCA), Breast Invasive Carcinoma (BRCA), Cervical Squamous Cell Carcinoma and Endocervical Adenocarcinoma (CESC), Cholangiocarcinoma (CHOL), Colorectal Adenocarcinoma (COADREAD), Diffuse Large B-Cell Lymphoma (DLBC), Esophageal Carcinoma (ESCA), Glioblastoma Multiforme (GBM), Head and Neck Squamous Cell Carcinoma (HNSC), Kidney Chromophobe (KICH), Kidney Renal Clear Cell Carcinoma (KIRC), Kidney Renal Papillary Cell Carcinoma (KIRP), Acute Myeloid Leukemia (LAML), Brain Lower Grade Glioma (LGG), Liver Hepatocellular Carcinoma (LIHC), Lung Adenocarcinoma (LUAD), Lung Squamous Cell Carcinoma (LUSC), Mesothelioma (MESO), Ovarian Serous Cystadenocarcinoma (OV), Pancreatic Adenocarcinoma (PAAD), Prostate Adenocarcinoma (PRAD), Sarcoma (SARC), Skin Cutaneous Melanoma (SKCM), Stomach Adenocarcinoma (STAD), Thyroid Carcinoma (THCA), Thymoma (THYM), Uterine Corpus Endometrial Carcinoma (UCEC), Uterine Carcinosarcoma (UCS), and Uveal Melanoma (UVM).

To group genes into pathways, we combined several databases of genes activated or repressed as a result of an activation of signaling pathway (pathway downstream genes): SPEED, PROGENy, Duke University and Curie Institute-curated data sets (Martignetti et al. (2016); Gatza et al. (2010); Parikh et al. (2010); Rydenfelt et al. (2020); Schubert et al. (2018)). Merging these databases resulted in a total of 69 unique sets of pathway downstream genes, which were further used in our study.

Of note, we made a choice to use in this study only genes representing downstream targets of signaling pathways instead of other available gene sets representing pathway players, *e.g.,* Reactome or KEGG (Fabregat et al. (2018); Kanehisa and Goto (2000)), or biological processes from Gene Ontology (Ashburner et al. (2000)) since biologically gene expression of pathway downstream genes only is expected to show coordinated changes.

### 2.2 Group Lasso

The Cox proportional hazards model is the most common survival prediction model for cancer prognosis. We denote *m* the number of covariates (genes) and *n* the number of patients. Moreover, $x=\left(x_{1},\ldots ,x_{n}\right)\in R^{m,n}$ is the (standardized) gene expression data matrix. For each patient, *Y*_*i*_ is the time of event, *i* = 1, … , *n*, and *C*_*i*_ is its type: *C*_*i*_ = 1 stands for deceased and *C*_*i*_ = 0 for right-censored (removed from study) patients. The negative log-partial likelihood associated with the Cox model is then defined as

$$\ell \left(\beta \right)=-\underset{i:C_{i}=1}{\sum }\left(x_{i}\cdot \beta -\log\underset{j:Y_{j}\geq Y_{i}}{\sum }e^{x_{j}\cdot \beta }\right),$$ (1)

where $\beta \in R^{m}$ is the (unknown) dependence of patients’ survival on their gene expression: positive elements correspond to the positive association of gene expression with a poor prognosis.

We are interested in ***β*** minimizing *ℓ*(***β***) in (1). The minimum is, however, not well defined for *m* ≫ *n*, which is often the case in the cancer survival analysis setting. Tumor databases typically include several hundreds of patients characterized for over 20,000 gene expression values. A remedy is provided by adding a regularization term, the most popular being ridge and lasso, or their combination into an elastic net (Zou and Hastie (2005)). In this work, we use the standard lasso term penalty

$$P_{\lambda }\left(\beta \right)=\lambda \|\beta {\|}_{1},$$ (2)

where *λ* is a non-negative constant corresponding to the strength of the regularization. Finding ***β*** that minimizes

$$\ell \left(\beta \right)+P_{\lambda }\left(\beta \right)=-\underset{i:C_{i}=1}{\sum }\left(x_{i}\cdot \beta -\log\underset{j:Y_{j}\geq Y_{i}}{\sum }e^{x_{j}\cdot \beta }\right)+\lambda \|\beta {\|}_{1}$$ (3)

produces a sparse solution where some of the coefficients are reduced to zero. However, while such regularization usually improves survival predictions, one of the important limitations remains excessive variation in selected genes across models trained on even slightly varying data (*e.g.*, different folds in a cross-validation).

In addition to the classic lasso setting, here we explore the group lasso model, where genes are grouped by molecular pathways. However, two distinct pathways often share a number of common genes. In the standard group lasso setting each gene only has a single coefficient and thus if a gene is truncated in one pathway it will be truncated in all of them. However, a simple duplication of genes occurring in two or more pathways has been shown to solve this issue and is known as latent group lasso (Jacob et al. (2009); Obozinski et al. (2011)). Therefore, we consider pathways as non-overlapping; but the overall gene set contains repetitive elements.

More precisely, we have a partition of the index set {1, … , *m*} into non-overlapping sets (groups). Consider a group *g* and $u=\left(u_{1},\ldots ,u_{m}\right)\in R^{m}$ . Then $u_{g}\in R^{m}$ denotes its projection to $R^{\left|g\right|}$ : ${\left(u_{g}\right)}_{i}=u_{i}$ for *i* ∈ *g*, and ${\left(u_{g}\right)}_{i}=0$ otherwise. Here, |*g*| is the number of elements in group *g*. In this work, we use the latent group lasso constraint

$$R_{\lambda }\left(\beta \right)=\lambda \underset{g}{\sum }\sqrt{\left|g\right|}\|\beta_{g}{\|}_{2}.$$ (4)

The Cox group lasso regression then will minimize the following loss function:

$$\ell \left(\beta \right)+R_{\lambda }\left(\beta \right)=-\underset{i:C_{i}=1}{\sum }\left(x_{i}\cdot \beta -\log\underset{j:Y_{j}\geq Y_{i}}{\sum }e^{x_{j}\cdot \beta }\right)+\lambda \underset{g}{\sum }\sqrt{\left|g\right|}\|\beta_{g}{\|}_{2}.$$ (5)

**FIGURE 1:**
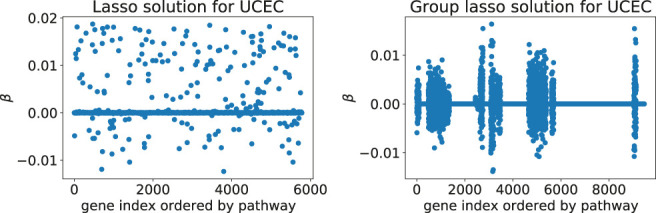
Typical solutions minimizing the Cox loss function with either the lasso **(left)** or the group lasso regression term **(right)**, here computed for the UCEC gene expression data: these two models predict survival of cancer patients based on expression values for genes from downstream targets of 69 signaling pathways. The number of inputs varies between the two examples since the implementation of latent group lasso duplicates genes that appear in more than one group (*i.e.*, set of signaling pathway downstream genes).

Adding *R*_*λ*_(***β***) to the loss function *ℓ*(***β***) effectively shrinks some of the coefficient groups to 0. Hence, one obtains a sparse model where only some of the covariate groups have non-zero coefficients (Figure 1).

Since many genes have correlated expression, the full set of genes is generally not necessary to achieve a good model accuracy. Typically, the group lasso is expected to achieve a similar precision as the standard lasso; however, we hypothesize that it will provide both better interpretability as well as higher congruence across folds. Since our gene grouping is based on cancer associated signaling pathways, the selected groups should be informative of cancer driving molecular processes.

### 2.3 Multi-Task Model

The single-type cancer survival prediction accuracy can be limited by various factors, *e.g.*, the low number of patients, noise, or high proportion of censored patients. The goal of the multi-task model that we introduce here is to improve that accuracy by forcing sharing (with some re-scaling coefficients) ***β*** weights of gene contributions to survival between different cancer types. We design a penalty for coupling gene contributions in a per-pathway way, assuming that gene contributions to pathway activities should be constant and therefore gene contributions to survival, which is driven by pathway deregulations, should be proportional across cancer types.

Let us consider two cancers with their corresponding loss functions $\ell \left(\beta^{j}\right)+R_{\lambda_{j}}\left(\beta^{j}\right)$ , *j* = 1, 2. To force a coupling between the coefficients ***β*** ^1^ and ***β*** ^2^, we introduce a new penalty term:

$$C_{\mu }\left(\beta^{1},\beta^{2}\right)=\mu {\left(\underset{g}{\sum }|g|{\left(A_{g}^{12}+A_{g}^{21}\right)}^{2}\right)}^{1/2},\;\text{where}\;A_{g}^{ij}=\left\|\left(\beta_{g}^{i}-\beta_{g}^{j}\frac{\left\|\beta_{g}^{i}\right\|}{\left\|\beta_{g}^{j}\right\|}\right)I_{\beta_{g}^{i}}I_{\beta_{g}^{j}}\right\|.$$ (6)

Here, *μ* is a hyperparameter corresponding to the strength of the coupling term *C*_*μ*_(***β*** ^1^, ***β*** ^2^) and *I* denotes the indicator function.

The penalty *C*_*μ*_ has the following properties:

1) for each pathway *g* actively contributing to patients’ survival, the penalty matches $\beta_{g}^{1}$ and $\beta_{g}^{2}$ ,
2) normalization with $\left\|\beta_{g}^{i}\|/\|\beta_{g}^{j}\right\|$ allows for matching in a situation when the same pathway is differentially predictive for survival in two cancers,
3) if a pathway is not important for patients’ survival in one of the cancers, the indicator function will remove corresponding coefficients from the matching penalty, and
4) the penalty is symmetric.

Finally, we find ***β*** ^1^ and ***β*** ^2^ minimizing the following loss function to produce maximum partial likelihood estimates of the model parameters:

$$\ell \left(\beta^{1}\right)+R_{\lambda_{1}}\left(\beta^{1}\right)+\ell \left(\beta^{2}\right)+R_{\lambda_{2}}\left(\beta^{2}\right)+C_{\mu }\left(\beta^{1},\beta^{2}\right).$$ (7)

The loss function (7) can be extended to an arbitrary number *k* of cancer types. Note that the number of hyperparameters is growing quadratically since there are *k* terms *R*_*λ*_, and *k*(*k* − 1)/2 terms *C*_*μ*_.

### 2.4 Assessing Model Accuracy and Reproducibility

We define a hazard score **x**_*i*_ ⋅ ***β*** for each patient *i* = 1, … , *n*. In this work, we used the *concordance index* (*c*-value) on the test data to evaluate model accuracy (Steck et al. (2008)). The *c*-value is equal to the proportion of pairs of observations where an event occurred first for an individual with a higher hazard score predicted by the model.

The interpretability of the model is conditional on how consistent the pathway selection is over different random seeds. As a measure of consistency, we compute the Tucker’s congruence coefficient (Tucker (1951)), and average it over all pairs of ***β***. To assess its significance, we carry out a paired *t*-test over the congruence of non-overlapping pairs of ***β***.

### 2.5 Model Optimization

To find ***β*** minimizing the loss functions of lasso, group lasso and multi-task group lasso models, we used the Adam optimizer implemented in the PyTorch package (Kingma and Ba (2014)). Moreover, in case of group lasso or multi-task group lasso, we truncated ***β***_*g*_ to zero when all elements from a group *g* were below a threshold of 0.001 in absolute value.

#### Selection of Hyper-Parameters

For each cancer type, we selected the hyperparameter *λ* using a 10-fold cross-validated grid search over a suitable range on the training set. We then performed 100 random 80–20 training-test splits, computed ***β*** on the training sets and evaluated the *c*-value on the test sets. Finally, we computed the paired *t*-test statistics value and its associated *p*-value, along with a congruence coefficient for both lasso and group lasso cases.

In the multi-task setting, along with *λ*_1_ and *λ*_2_ parameters, we select the best value of the coupling parameter *μ*, which we do in a similar cross-validation loop as for the standard lasso and group lasso. With a growing number of tasks, a grid search over multiple hyperparameters becomes computationally demanding or even unfeasible. An implementation of a random search then provides a possible solution. To determine *λ*_*j*_ in the multi-task setting, 30 values were selected randomly from a normal distribution with the mean set as the *λ*_*j*_ previously calculated from standard group lasso and a standard deviation of 0.1*λ*_*j*_. Additionally, 30 values for *μ* were randomly selected from a half-normal distribution around 0 with standard deviation 0.5 (chosen heuristically). By selecting the best cross-validated set of hyperparameters per task, in the Results section, we compared the performance (*c*-values) of the multi-task model with its single-task counterpart.

### 2.6 Training and Testing a Multi-Task Model

#### Training on Synthetic Data

To check the validity of our multi-task learning approach and corresponding code, we simulated the following synthetic data set: Two “toy” cancer gene expression and survival data sets *T*_1_ and *T*_2_ drawn from a normal sampling distribution generated from two TCGA cancers COADREAD and STAD. Both *T*_1_ and *T*_2_ comprised nearly 10,000 genes, and 300 and 200 patients respectively. Moreover, we assumed that the patients’ survival is fully determined by two pathways each where one is being shared among the two toy cancer types. The corresponding “true” ***β*** coefficients were obtained as the first principal component coefficients of the genes included in the pathway over the combined COADREAD and STAD data sets.

To each patient *i*, we randomly assigned either event *C*_*i*_ = 1 (with probability 70%) or censorship *C*_*i*_ = 0 (30%). The score ***x***_*i*_ ⋅ ***β*** is an indicator of the patient’s risk. In case all patients were deceased, we could use − **x** ^*T*^ ***β*** as the time-of-event **Y** (since actual values do not matter in the Cox model (1), only their ordering). However, since censorship only provides a lower bound on the time of death, we randomly decreased the censored patients’ times *Y*_*i*_ as a function of the number of patients with a higher score.

We trained individual latent group lasso and multi-task models. After hyperparameter selection, 100 80–20 splits were performed to calculate significance.

#### Training on TCGA Data

We examined all possible pairs between 30 cancer types in the TCGA data set. For each pair, we selected hyperparameters using a 10-fold cross-validated random search. We then performed 30 80–20 training-test splits, computed ***β*** on the training sets and evaluated the *c*-value on the test sets for both cancers. We computed the paired *t*-test statistics value and its associated *p*-value for each pair with respect to the latent group lasso without multi-tasking. Finally, the false discovery rate (FDR) correction for the number of pairs tested per cancer was applied.

## 3 Results

### 3.1 Latent Group Lasso

**FIGURE 2:**
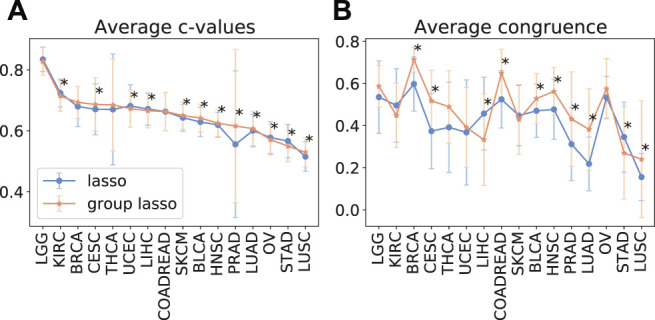
The average test prediction accuracy (*c*-value, **(A)**) and model stability (average congruence coefficients, **(B)**) for the standard and group lasso models, evaluated on 16 cancers from the TCGA data set. The error bars represent standard deviations. The asterisks (*) mark significant *p*-values at the *p* <0.05 level.

**FIGURE 3:**
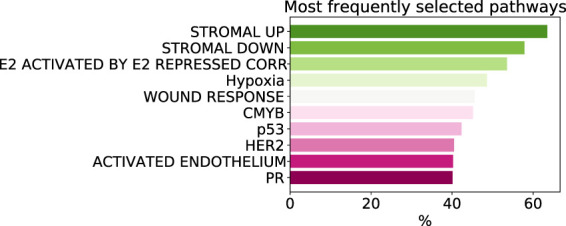
The most commonly selected pathways by group lasso across 16 cancer types with more that 300 samples. The frequency is computed over all random tests, totalling 1,600 data points.

As despite the popularity of group lasso, we could not find a comparison between standard lasso and group lasso model for the cancer survival prediction on gene expression data, we first evaluated and compared accuracies of these two models on 16 cancer types from the TCGA database with at least 300 patients per set (see Section 2.1 for data set description). Out of the 16 cancers tested, five had a significantly higher prediction accuracy (*c*-value) for simple lasso, seven were significantly higher for latent group lasso and there was no significant difference for the remaining four cancers (Figure 2A). Also, model reproducibility measured through the averaged congruence coefficients (see Section 2.4) was better for the group lasso model for 12 out of the 16 cancers tested (Figure 2B). The most frequently selected pathways across all cancer types over all random tests (*i.e.,* 16 × 100 data points) are plotted in Figure 3. We observed that the most common pathways are the stromal up-(63%) and downtake (58%).

Our results showed a very modest improvement in prediction accuracy from applying latent group lasso to cancer survival; however, we hypothesized that this accuracy could be improved by adding a multi-task term to the loss function to allow sharing information across cancer types.

### 3.2 Validating the Multi-Task Penalty on Synthetic Data

**FIGURE 4:**
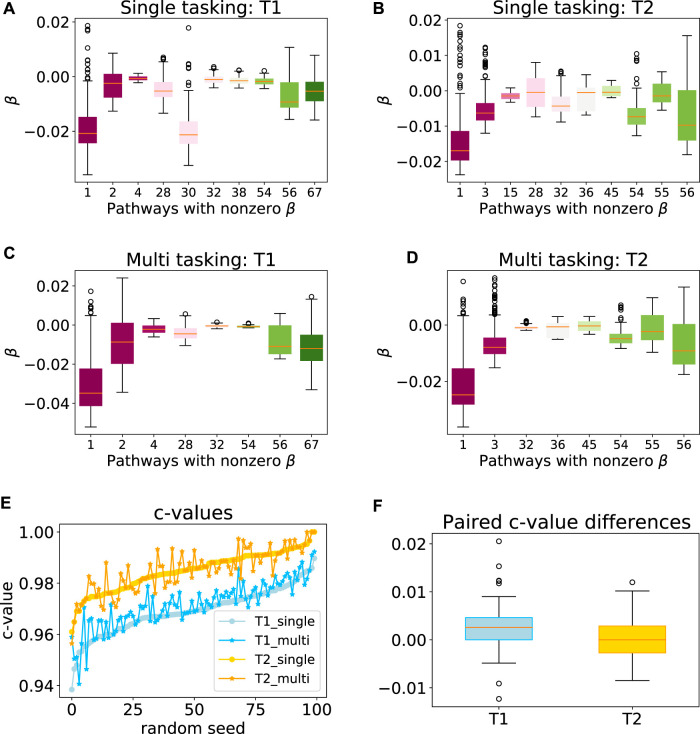
Performance of the multi-task model on a synthetic data set. Box plots showing the range of model coefficients ***β*** by pathway for standard group lasso **(A–B)** and multi-task group lasso **(C–D)** on *T*_1_ and *T*_2_ data sets. Out of the 69 total pathways, only pathways with at least one non-zero coefficient are shown. By design, activity of pathways 1 and 2, and one and three were predictive for patient survival for *T*_1_ and *T*_2_, respectively. The average congruence coefficient of ***β*** is 0.81 (*T*_1_) and 0.96 (*T*_2_) for single tasking, and 0.80 (*T*_1_) and 0.91 (*T*_2_) for multitasking (e–f) *c*-values and box plots of the paired difference for 100 random seeds for single and multi-tasking for *T*_1_ and *T*_2_ synthetic data. Note that *T*_1_ and *T*_2_ are sorted independently, so their random seed numberings do not correspond. The *t*-test *p*-values are 1.35⋅10^–7^ (*T*_1_) and 0.49 (*T*_2_).

To explore the efficacy of the multi-task penalty (7) we designed, we first applied our approach to synthetic data sets *T*_1_ and *T*_2_ comprising 300 and 200 samples respectively (see Section 2.6 for the detailed data set description). Our simulation results showed that while the latent group lasso without multi-tasking generally selected the correct pathways for *T*_1_ (pathways 1, 2) and *T*_2_ (pathways 1, 3) the model also assigned non-zero coefficients to a number of the irrelevant pathways (Figures 4A,B). However, when the multi-task penalty was added, the number of irrelevant pathways included in the model usually reduced for both data sets (Figures 4C,D), and no correctly included pathways were lost. Furthermore, the average *c*-value increased significantly for *T*_1_ when the multi-task penalty was included, and did not change significantly for *T*_2_ (Figures 4E,F). From these results, we concluded that the multi-task penalty we designed was acting as intended. Finally, however, the congruence of the models across folds decreased for both sets, significantly for *T*_2_.

### 3.3 Multi-Task Group Lasso Model on the TCGA Data

**FIGURE 5:**
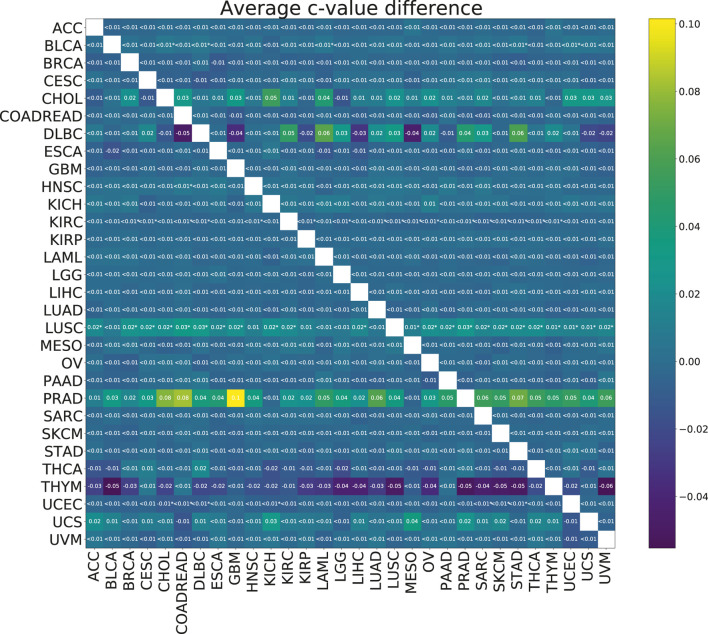
Heatmap showing the mean paired difference in *c*-value between single and multi-task training. Positive values correspond to the improvement of model prediction accuracy with multi-tasking. The rows correspond to cancer types for which the *c*-values were calculated and the columns to cancer types with which the target cancer was paired for the multi-task training. Asterisks (*) indicate significance with the FDR corrected *p*-value *p* <0.05.

To check the efficacy of the multi-task group lasso model for the survival prediction, we applied it to 30 TCGA cancer data sets (see Section 2.1 for more details). For each pair of cancer types, we compared the resulting model accuracies (*c*-values) calculated for 100 random splits for the individual group lasso and multi-task group lasso models. Although based on the results of the model validation on synthetic data, we expected the multi-task setting to improve predictions, little significant difference was observed after multiple testing correction (Figure 5). For one cancer type, LUSC, significant improvements were observed when the cancer was paired with a number of other cancer types. Further, while not significant after multiple testing correction, significant uncorrected differences were observed for PRAD. In particular, combining PRAD with CHOL, COADREAD and GBM each led to an improvement of *c*-value over 0.08. Finally, several other combinations showed a marginal significant improvement, *e.g.*, BLCA with STAD, KIRC *w*th KIRP, or UCEC with COADREAD.

**FIGURE 6:**
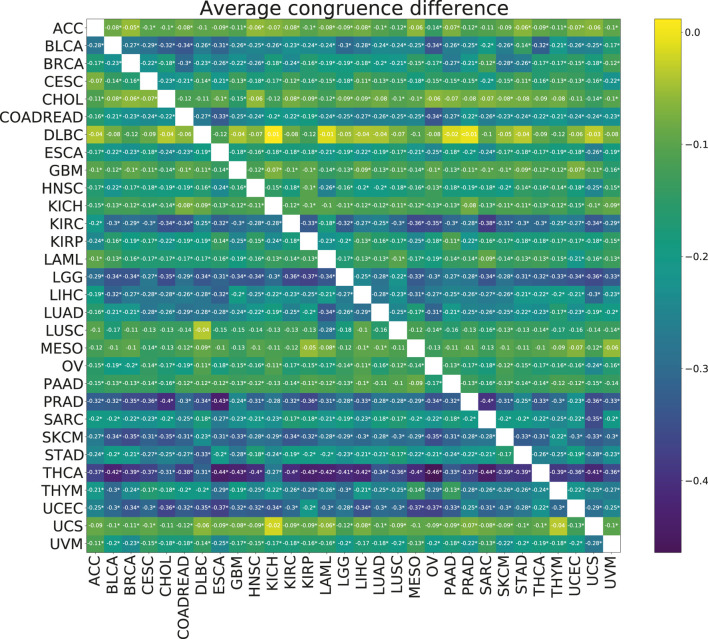
Heatmap showing the mean paired difference in congruence between single and multi-task training. Positive values correspond to the improvement of model stability with multi-tasking. The row gives the cancer for which the congruence was calculated and the columns give the cancer with which the target cancer was paired for the multi-task training. Asterisks (*) indicate significance with the FDR corrected *p*-value *p* <0.05.

No significant improvements in the model stability, measured by congruence between model coefficients ***β***, were observed with the addition of multi-tasking (Figure 6). The mean congruence decreased for almost every cancer pair tested.

Of note, other multi-tasking approaches using the same data and similar validation strategies, such as VAECox (Kim et al. (2020)), have reported similar results, with only limited improvements over standard lasso. VAECox observed a microaverage concordance across 10 cancers from TCGA of 0.649; using the same microaverage method for those 10 cancers, our multi-task approach gave results in the range 0.645–0.663, depending on the paired cancer type.

## 4 Discussion

In this paper, we assessed the efficacy of different regularization penalties for linear models for survival prediction on cancer gene expression data. First, we compared standard lasso with latent group lasso. This analysis showed a very slight overall improvement in survival prediction accuracy when using molecular pathways as *a priori* known groups compared to simple lasso. In short, for seven cancers the prediction accuracy significantly increased, significantly reduced for five cancer types, and for the remainder it did not significantly vary between the two methods. This suggested that latent group lasso alone does not meaningfully improve cancer survival predictions beyond what can be achieved with naïve lasso when using gene expression data. Despite these modest results, we observed that model stability, *i.e.*, congruence between model coefficients when training using different random seeds, appeared to be higher for latent group lasso regularization, suggesting potential improvements in biological interpretability.

Next, we tested our multi-tasking model a on syntetic data set designed so that it closely mimicked real cancer data (including strong gene collinearity). We used two toy sets drawn from a sample distribution associated with COADREAD and STAD, and then determined the patients’ hazard scores from two overlapping gene groups each. We randomly censored 30% of the patients and adjusted for their survival time uncertainty. In order to leverage similarities between cancers, we introduced a rather low number of patients–300 and 200 respectively. Our model showed a comparably high *c*-value for both toy cancers separately, and a significant improvement in the accuracy of the first set after multi-tasking. Moreover, fewer irrelevant pathways were generally selected with multi-tasking compared to the univariate model, though the congruence decreased, significantly for the second data set. Therefore, we would expect similar improvements in real data sets, especially if they comprise a low number of patients.

However, in the multi-tasking test on experimental data, we saw relevant significant improvements in prediction accuracy measured by *c*-value with only one cancer type, LUSC. For this type of cancer, we witnessed extremely poor performance of single-task group lasso regression on gene expression data, generally giving results around 0.52 of *c*-value, marginally above the random level (0.5). This value improved slightly with multi-tasking up to 0.53. Further, we observed the largest, albeit not significant improvement in *c*-value for PRAD. However, the comparably high survival rate (10 deaths for 498 patients) causes a large variance in the *c*-values due to the random fold splitting. The improvement in *c*-value for both LUSC and PRAD occurred when they were paired with many different cancers and the improvements were of a similar magnitude across the board. This suggested that the benefit here was not from finding a similar cancer to leverage from but more that any extra available information was benefitting survival models, which are inherently difficult to build from expression data. Our initial intuition that survival models for cancer types sharing similar features, such as ovarian and cervical cancers or uveal and skin melanomas, would benefit from multi-tasking was not confirmed.

We hypothesize that this may depend on the noise in the data and measurement uncertainties, or simply the limitation of gene expression prediction power. We cannot exclude however that different, possibly non-linear cancer survival models could benefit from multi-tasking and prior knowledge on pathway downstream genes. We are going to explore this type of approaches in our future work.

For our linear group lasso-based approach, we also tested a number of other potential coupling penalty terms, including very simple ones such as penalizing the mean absolute difference in coefficients (Table 1). None of these approaches were as successful on our synthetic data as the one that has been presented in this work, but we include them for completeness.

**TABLE 1:** Alternative coupling penalty terms that were given preliminary investigation using synthetic data.

Coupling term	Preliminary results
$\mu \sum_{g}\sqrt{\left|g\right|}\left\|\beta_{g}^{1}-\beta_{g}^{2}\right\|$	This term was discarded as it did not allow for different scaling for $\beta_{g}^{1}$ and $\beta_{g}^{2}$ between cancer types
$\mu \sum_{g}\sqrt{\left|g\right|}\left|1-\frac{\beta_{g}^{1}\cdot \beta_{g}^{2}}{\left\|\beta_{g}^{1}\|\;\|\beta_{g}^{2}\right\|}\right|$	This term allowed matching of $\beta_{g}^{1}$ and $\beta_{g}^{2}$ as intended, but did not show improvement of *c*-value on synthetic data
$\mu \sum_{g}\sqrt{\left|g\right|}\left\|\frac{\beta_{g}^{1}}{\left\|\beta_{g}^{1}\right\|}-\frac{\beta_{g}^{2}}{\left\|\beta_{g}^{2}\right\|}\right\|\sqrt{\left\|\beta_{g}^{1}\right\|}\sqrt{\left\|\beta_{g}^{2}\right\|}$	This term allowed matching of $\beta_{g}^{1}$ and $\beta_{g}^{2}$ as intended and showed improvement of *c*-value on synthetic data. However, the improvement was slightly worse than for the penalty we proposed in (7) and used in this study

Although theoretically our approach could be extended to triplets of cancer types and larger groups, we do not present these results here. Indeed, several tests applied on cancer triplets did not show strong positive results, which was expected given moderate performance of our new model on cancer pairs.

To sum up, in this study we addressed the question of building cancer survival models on gene expression data when incorporating both information about pathway downstream genes and multi-tasking across different cancer types. For the majority of cancer types we tested, the performance of our multi-task model was generally comparable with that of the latent group lasso and classic lasso approaches. However, we would advocate for the use the individual latent group lasso because of the improved model stability and interpretability.

## Data Availability

The code to run multi-task group lasso on the Tumor Genome Atlas (TCGA) and synthetic data sets is available at https://github.com/BoevaLab/Group_Lasso_and_Multitask.
